# External validation, recalibration and updating of the OxSATS risk model for suicide after self-harm in England

**DOI:** 10.1136/bmjment-2026-302568

**Published:** 2026-09-12

**Authors:** Tyra Lagerberg, Denis Yukhnenko, Maria D L A Vazquez-Montes, Thomas R Fanshawe, Seena Fazel

**Affiliations:** 1 Department of Psychiatry, University of Oxford, Oxford, UK; 2 Nuffield Department of Primary Health Care Sciences, University of Oxford, Oxford, UK

**Keywords:** Suicide, Self-harm, Risk assessment, Prediction models

## Abstract

**Background:**

External validations of existing risk models are efficient steps towards potential implementation, avoiding the need to develop new models. However, validation in new clinical settings poses several challenges.

**Objective:**

To externally validate the OxSATS tool using data from the Oxford Monitoring System for Self-harm in England. OxSATS is a validated tool to predict suicide after self-harm developed using Swedish population registers.

**Methods:**

We selected episodes of self-harm (International Classification of Diseases, Tenth Revision codes X60–X84; Y10–Y34) by individuals who presented to a large regional hospital between 1 January 2000 and 31 December 2018, and were followed up until 31 December 2019. We applied the OxSATS tool to estimate each individual’s suicide risk within 12 months after their index self-harm. We assessed model performance using discrimination (Harrell’s concordance index (c-index)) and calibration measures (calibration plot and the observed-to-expected events ratio (O:E)). We assessed the effects of missing predictors on calibration and subsequently recalibrated the model.

**Findings:**

We identified 16 130 individuals who presented to hospital with self-harm, of whom 106 (0.7%) died by suicide in the 12-month follow-up period. The OxSATS model showed good discrimination in external validation (c-index 0.73, 95% CI 0.68 to 0.79). Recalibration was required because initial calibration reflected a lower outcome rate in the new data. After recalibration, calibration performance was excellent (O:E 1.00, 95% CI 0.77 to 1.25).

**Conclusions:**

Despite differences in clinical services and outcome ascertainment, suicide risk models can maintain good predictive performance in new settings. However, recalibration should be considered when applying prediction models in new settings, and the impact of missing predictors should be assessed using sensitivity analyses.

**Clinical implications:**

OxSATS may help clinicians assess suicide risk after self-harm by providing structured and transparent estimates as part of a comprehensive clinical assessment. Structured risk assessment tools may support identification of individuals who could benefit from additional risk management procedures, such as closer monitoring and follow-up or referral to specialist services, particularly in settings with limited access to such services.

**WHAT IS ALREADY KNOWN ON THIS TOPIC**

- Suicide risk is substantially elevated after hospital presentation for self-harm, but most existing risk assessment tools rely on rating scales or binary cut-offs, show limited predictive accuracy and rarely report calibration.
- The Oxford Suicide Assessment Tool for Self-harm (OxSATS) is a prognostic model developed using Swedish register data that provide continuous risk estimates and demonstrated good discrimination and calibration in its original setting.
- External validation in new healthcare systems is considered good practice before implementation, but is often complicated by differences in predictor definitions, missing variables and outcome prevalence.

**WHAT THIS STUDY ADDS**

- This study provides the first external validation of OxSATS in an English clinical setting using routinely collected hospital data.
- The model retained good discrimination but initially overpredicted suicide risk due to a lower baseline event rate and one missing predictor, highlighting the importance of calibration assessment.

**HOW THIS STUDY MIGHT AFFECT RESEARCH, PRACTICE OR POLICY**

- Where feasible, future research and implementation strategies could incorporate external validation, sensitivity analyses for missing predictors, and local recalibration before clinical or policy adoption.

## Introduction

 Suicide is a leading cause of death, particularly among children and young adults.^1^ A major risk marker for suicide is prior self-harm—between 1% and 2% of those who present to clinical services with self-harm go on to die by suicide over the next 12 months.^2 3^ Identifying individuals at particularly elevated risk in this population can potentially inform decision-making around risk management, resource allocation and follow-up.

The Oxford Suicide Assessment Tool for Self-harm (OxSATS) was developed using Swedish national population registers to estimate suicide risk after presentation with self-harm to emergency departments and secondary mental health services. The risk model showed good predictive performance in Swedish validation in relation to discrimination (Harell’s concordance index (c-index) 0.77, 95% CI 0.75 to 0.78) and calibration (observed-to-expected events ratio (O:E) 0.92, 95% CI 0.79 to 1.06) and provides a continuous risk score for flexible application in research and clinical care. The output of continuous probability scores is a key difference to previous tools, which have relied on binary cut-offs and evaluation of performance only on classification measures (such as sensitivity and specificity) while neglecting to consider model calibration and overall discrimination. Another difference is that almost all previous tools used in practice are rating scales not developed or intended for prognostic probability prediction.^3^ OxSATS, in contrast, was developed using high-quality methods for prediction modelling, including a prespecified analytic protocol, empirically derived predictors, clear prediction horizons and a large population dataset, ensuring outcome numbers sufficient for model development. Predictors were chosen to be generalisable to different settings. Reporting of the model was comprehensive and transparent, following established guidelines.

Nevertheless, implementation of OxSATS for research, training and clinical purposes in settings outside Sweden should consider testing its predictive performance in the new intended population (external validation) and recalibrate the model if poor calibration is demonstrated. In a new country or setting, using a tool already developed is an efficient research approach, as it does not require investment in developing a new tool, which requires larger data sets.^4^ We therefore aimed to test the performance of the OxSATS risk model using data from a regional database in England, the Oxford Monitoring System for Self-harm. Validating prediction models in new settings is associated with challenges, including different predictor definitions, missing predictors, and a base event rate and outcome definition that can vary from the original model development sample.

## Methods

### Data sources

The Oxford Monitoring System for Self-harm contains data of individuals who presented to an emergency department with a non-fatal self-harm event at a large general hospital in Oxford.^5^

### Study population

We included individuals aged 10 years or above who presented to an emergency department with an episode of self-harm between 1 January 2000 and 31 December 2018. In individuals with more than one self-harm episode, we randomly selected a single episode as the index, in keeping with previous work and the original model development.^3^ This approach avoids systematic bias towards earlier or later episodes and better reflects how the tool would be applied in practice, where individuals can be assessed at different time points rather than only at first presentation.

We followed up individuals for 12 months up to 31 December 2019, from the date of their index self-harm episode to determine whether they died by suicide during that period. As a sensitivity analysis, we repeated analyses using alternative index episode definitions (first and most recent episode) to assess the robustness of findings.

### Prediction model

The OxSATS risk model was developed and validated in 53 172 individuals aged at least 10 who presented to specialist services for non-fatal self-harm in Sweden between 2008 and 2012.^3^ It includes 11 routinely collected predictors and is available as an online risk calculator at https://oxrisk.com/oxsats/.

### Definition of outcomes

The primary outcome was death by suicide within 12 months of presentation with non-fatal self-harm to specialist services, defined using International Classification of Diseases, Tenth Revision ICD-10 codes X60–X84 (intentional self-harm), consistent with the original model.^3^

As part of sensitivity analyses, we additionally examined an alternative outcome definition that included deaths of undetermined intent (ICD-10 Y10–Y34) to assess the impact of potential misclassification and underascertainment. We additionally examined the outcomes over the extended follow-up period of 3 years.

### Definition of predictors

Predictor information was collected within the Oxford Monitoring System for Self-harm, which is closely integrated with the Emergency Department Psychiatric Service at the study hospital. Data are primarily recorded by clinicians during psychosocial assessments using structured forms, supplemented by emergency department records for individuals not receiving a psychosocial assessment. The variables used in this study therefore reflect routinely collected clinical information recorded at the time of presentation, based on clinician assessment and available records, rather than data collected specifically for research purposes. Values coded as ‘not known’, ‘not collected’ or equivalent non-informative responses were recoded as missing.

All predictors in OxSATS were present or had an equivalent in the Oxford Monitoring System for Self-harm data apart from “any psychotropic medication receipt [collection of a prescription] in past 3 months” (current psychotropic medication use). The predictors included age, sex, current or lifetime alcohol use disorder, current or lifetime drug use disorder, prior self-harm (psychotropic drug overdose; any prior hanging, strangulation or suffocation and any prior self-harm within 12 months of the index episode) and any psychiatric disorder except for substance use disorder. The covariates were collected at the time of admission by clinicians, were assumed to be complete and no missing data imputation was performed.

One predictor from the original model was not available, which was current use of any psychotropic medication. We accounted for the impact of this missing predictor through sensitivity analyses assuming different prevalence levels in the population (see ‘Statistical methods’ section). We compared prevalence of predictors available in the OxSATS development sample and Oxford Monitoring data (see ‘Results’ section). One difference in definitions was that the OxSATS predictors for psychiatric diagnoses were based on lifetime diagnoses, while the equivalent predictors in Oxford Monitoring data were coded as mental health conditions that healthcare staff judged as contributing to the individual’s presenting act of self-harm.

### Power analysis

To determine the required sample size, we used the *pmvalsampsize* package for R, following the methodology for precision-based power analysis for external validation studies.^6^ Assuming an outcome prevalence of 0.6% and anticipated discrimination consistent with OxSATS, a sample size of 16 130 individuals (approximately 97 outcome events) would be expected to provide the following precision in performance estimates: overall calibration (O:E 1.00, 95% CI 0.80 to 1.20), a calibration slope of 1.00 (95% CI 0.80 to 1.20) and discrimination of c-index 0.77 (95% CI 0.70 to 0.85). These calculations were used to characterise the expected precision of the validation analyses rather than to define a preregistered or fixed sample size target.

### Statistical methods

We tested predictive performance by estimating measures of discrimination and calibration. We used Harell’s c-index as a measure of discrimination. Calibration was assessed using calibration plots and the O:E ratio. As recommended by new Transparent Reporting of a multivariable prediction model for Individual Prognosis or Diagnosis-Artificial Intelligence (TRIPOD-AI) guidelines,^7^ we have not set predetermined thresholds for calibration and reported continuous calibration metrics. Calibration plots were created by plotting the mean predicted risk in 20 quantiles of the predicted probability against the proportion of observed events in each quantile.^8^

The primary analysis used complete-case data. As a sensitivity analysis, missing values in source variables (see online supplemental table S1 for missing data distribution) required to derive OxSATS predictors were handled using multiple imputation by chained equations prior to predictor derivation and risk-score calculation. Ten imputed datasets were generated and performance metrics obtained from them were combined using Rubin’s rules.^9^

For the predictor missing (current psychotropic medication use), we set its value as equal to its prevalence in the original OxSATS development sample, 0.557. As a sensitivity analysis, we tested the impact of randomly assigning the missing predictor as 0 or 1 in different individuals by using a Bernoulli distribution with p=0.557. We took the average of performance measures over 1000 Monte Carlo simulations. In further sensitivity analyses, we assessed model performance by setting the missing predictor to values ranging from 0 to 1 in steps of 0.1 to estimate how changes in the assumed probability influenced calibration.

We initially evaluated the performance of OxSATS without updating the formula for calculating predicted risk. If calibration was found to be poor, we planned to update the intercept, shape parameter and the predictors’ coefficients using a single multiplicative recalibration parameter. This procedure adjusts the calibration of the model while leaving the discrimination performance unchanged.^10^ We did not plan to re-estimate individual predictor coefficients as that would require creating a new prediction tool.

All analyses were carried out using Python V.3.9.11. We followed TRIPOD-AI guidelines on the reporting of validation studies (for the checklist, refer to online supplemental file 2).

## Results

We identified 16 130 individuals who presented to an emergency department in a regional hospital centre with self-harm (table 1, online supplemental table S2). The median age at the index self-harm episode was 27 years (SD 15.2), and 60% of individuals presenting with self-harm were female. A lifetime history of self-harm before the index episode was present in 44% persons, with 27% individuals having self-harmed in the 12 months preceding the index self-harm episode. Recorded mental health problems in the preceding 12 months were present in 15%. At index presentation, 29% involved an overdose with psychotropic medication, and 2% involved hanging, strangulation or suffocation.

**Table 1:** Prevalence of OxSATS predictors in the Oxford Monitoring System for Self-harm dataset, overall and stratified by whether individuals die from suicide within 1 year

Oxford Self-Harm monitoring	Oxford Self-harm monitoring study (n=16 130)	OxSATS (n=37 523)	OxSATS definition, if different from Oxford’s
Predictors			
Age, years: mean (SD)	32 (±15)	32.2 (-)	
Age, years: median (SD)	27.0 (20–40)	–	
Female	9625 (59.7%)	20 561 (54.8%)	
Problems* with alcohol	2622 (16.3%)	7257 (19.3%)	Current or lifetime alcohol use disorder diagnosis, excluding alcohol intoxication
Problems* with other substances	1119 (6.9%)	8384 (22.3%)	Current or lifetime drug use disorder diagnosis (including drug intoxication)
Any psychotropic medication receipt in past 3 months	–	20 888 (55.7%)	
Index self-harm: overdose with psychotropic medication	4740 (29.4%)	1360 (3.6%)	
Index self-harm: hanging, strangulation or suffocation	254 (1.6%)	308 (0.8%)	
Lifetime history of self-harm prior to index	7030 (43.6%)	11 277 (30.1%)	
History of self-harm in last 12 months prior to index	4288 (26.6%)	4281 (11.4%)	
Index self-harm: overnight admission	12 658 (78.5%)	16 991 (45.3%)	
Problems* with mental health	2350 (14.6%)	16 472 (43.9%)	Mental health diagnosis in last 12 months
Outcomes			
Suicide within 1 year of self-harm	106 (0.7%)	391 (1.0%)	
Suicide within 3 years of self-harm	164 (1.0%)	–	

* Problems=“the problem which may have precipitated the episode of self-harm or is associated with it in the clinician’s judgement”.

OxSATS, Oxford Suicide Assessment Tool for Self-harm.

In the Oxford Monitoring data, the outcome prevalence of death by suicide within 12 months of the index self-harm event was 0.7% when including deaths of undetermined intent (ICD-10 X60–X84, Y10–Y34), and lower at 0.5% when restricted to confirmed suicides only (ICD-10 X60–X84), compared with 1.0% in the OxSATS development sample. Several predictors also showed differences in prevalence between the samples. These included any psychiatric diagnosis (12.2% in Oxford Monitoring data vs 43.9% in the original OxSATS sample), problems with substances/substance misuse (6.9% vs 22.3%), and a history of self-harm (18.4% vs 11.4%).

In sensitivity analyses with extended follow-up (3 years), the outcome prevalence increased to 1.0% when including deaths of undetermined intent and 0.7% when restricted to confirmed suicides only.

Of the 16 130 individuals included in the analysis, 4581 (28%) had multiple self-harm episodes, while 11 550 (72%) had a single episode. A total of 31 649 episodes were available, with one randomly selected per individual for the main analysis. In sensitivity analyses, model performance was similar when defining the index episode as the first or the most recent episode, with comparable discrimination and calibration estimates (see online supplemental tables S3–S5).

Those who died by suicide within 12 months had a higher prevalence of all the predictors except problems with other substances; they were more likely to be male, have a history of self-harm, and overdose method of self-harm as part of their current presentation (online supplemental table S6).

**Figure 1:**
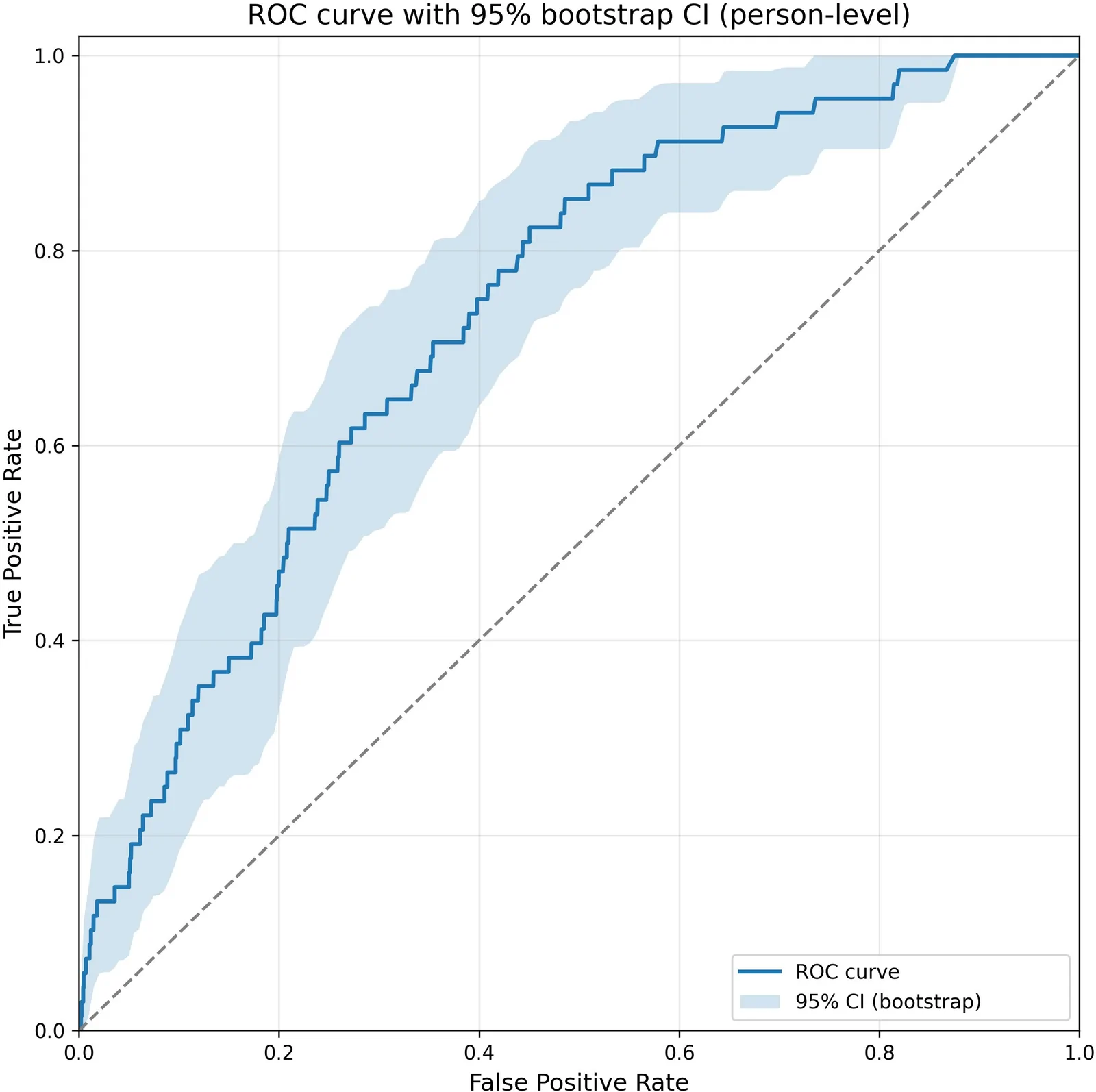
Receiver operating characteristics (ROC) curve of the Oxford Suicide Assessment Tool for Self-harm model in the Oxford Monitoring sample.

The discrimination performance of the OxSATS tool was good (c-index 0.73, 95% CI 0.68 to 0.79; area under the curve (AUC) 0.72, 95% CI 0.67 to 0.72) (table 2, figure 1). However, the uncalibrated OxSATS model overestimated the risk across predicted probabilities, resulting in poor calibration (O:E 0.57, 95% CI 0.47 to 0.69), with miscalibration being worse at higher probabilities (online supplemental figure S1).

**Table 2:** Discrimination performance in English compared with Swedish data

	Oxford self-harm data	OxSATS external validation sample
Suicide within 1 year prevalence (95% CI)	0.7% (0.6% to 0.8%)	1.1% (1.0% to 1.3%)
C-index (95% CI)	0.73 (0.68–0.79)	0.77 (0.75–0.78)

OxSATS, Oxford Suicide Assessment Tool for Self-harm.

Discrimination was consistent across sensitivity analyses. Estimates were similar when restricting the outcome to confirmed suicides only (ICD-10 X60–X84; c-index 0.76, 95% CI 0.70 to 0.81) and when defining the index episode as the first recorded self-harm episode (c-index 0.74, 95% CI 0.69 to 0.79). Calibration remained suboptimal across these specifications (O:E ranging from 0.41, 95% CI 0.30 to 0.53 to 0.56, 95% CI 0.43 to 0.70), indicating systematic overprediction.

The missing predictor (current psychotropic medication use) was set to 0.557 in initial model assessment to reflect the prevalence in the OxSATS development sample. In Monte Carlo simulation assigning 0 or 1 to each individual with p=0.557, the mean performance metrics over 1000 simulations were similar to the main analysis, with a mean O:E of 0.52 (95% CI 0.52 to 0.53) from the simulations, compared with 0.56 (95% CI 0.43 to 0.70) in the main analysis (online supplemental table S7). The O:E ratio decreased monotonically as the assumed prevalence of the missing predictor increased (online supplemental figure S2), indicating increasing overprediction at higher prevalence levels.

**Figure 2:**
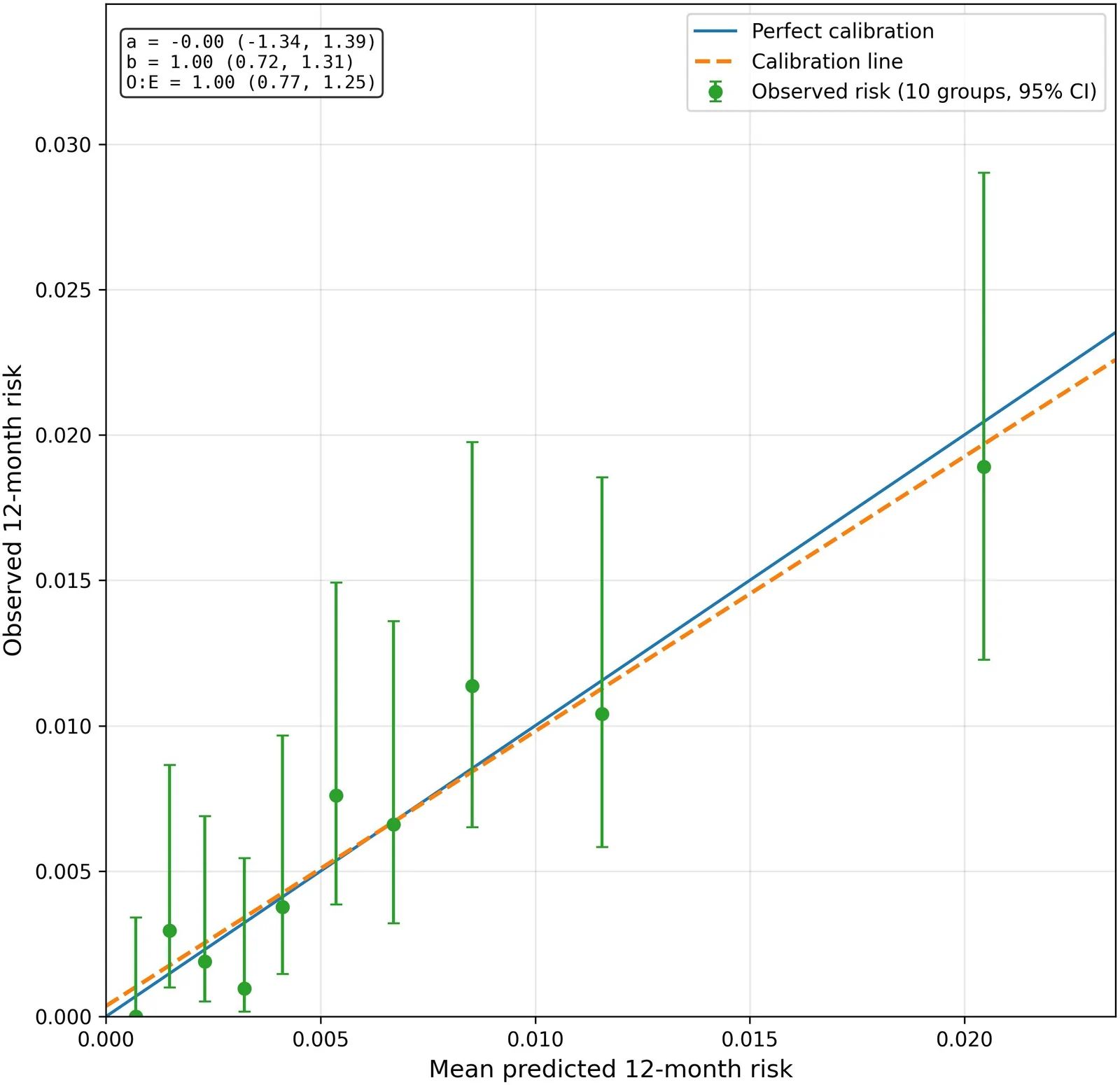
Calibration of Oxford Suicide Assessment Tool for Self-harm (OxSATS) model after recalibration. O:E, observed-to-expected events ratio.

We recalibrated the model by updating the intercept, shape parameter and applying a fixed multiplier for the linear predictor (online supplemental table S8). As expected, this resulted in a well-calibrated model (O:E 1.00, 95% CI 0.77 to 1.25; figure 2).

## Discussion

We investigated suicide mortality in 16 130 individuals who presented with a self-harm episode to a large regional English hospital. Over the follow-up of 12 months, 106 people died by suicide (0.7%). We tested the OxSATS tool to model suicide risk in the 12 months after the presenting self-harm episode and evaluated predictive performance using discrimination and calibration metrics. We found good overall discrimination performance (c-index 0.73, 95% CI 0.68 to 0.79). However, there was a lower suicide rate after self-harm in this study than in the Swedish development sample, which meant calibration was initially poor, with the OxSATS tool overpredicting suicide risk (O:E 0.56, 95% CI 0.43 to 0.70). Recalibration successfully aligned the expected event rate with the average observed risk in this cohort (O:E 1.00, 95% CI 0.77 to 1.25).

Estimating calibration thoroughly and considering recalibration is often overlooked as part of risk model validation.^11^ Unlike discrimination, which reflects only relative risk ranking, calibration determines whether absolute risk estimates generated by a risk model correspond to real-world event rates and allows for interpretation of model outputs and potential actionability. Poorly calibrated models can systematically overpredict or underpredict risk. Overprediction can lead to unnecessarily restrictive interventions such as overmonitoring or hospitalisation, while underprediction may result in insufficient follow-up and missed preventative opportunities. Ensuring good calibration is therefore essential for the safe and proportionate use of prediction models in clinical practice, especially for low-base-rate outcomes such as suicide.

### External validation and comparisons

Developing a new prediction tool for every new national context is costly and time-consuming—external validation of an existing tool offers an efficient use of resources. Additionally, there is often insufficient data to develop a prediction tool in new settings, particularly when considering rare outcomes. While we had adequate power over the 12-month and 3-year horizon, there was insufficient power to externally validate the OxSATS tool at the 6-month time horizon. Discrimination remained similar when extending the prediction horizon to 3 years, suggesting that the model captures stable relative risk differences rather than short-term risk alone.

Approximately 28% of individuals had multiple self-harm episodes, reflecting the recurrent nature of self-harm in this population. Although repetition is an important risk marker, model performance was robust to the choice of index episode, suggesting that the model captures underlying risk factors that generalise across different points in an individual’s clinical trajectory.

The results of this external validation show a reduction in performance compared with the original Swedish study (c-index of 0.73 vs 0.77), which would be expected given differences in national setting (England vs Sweden), data collection (questionnaires filled by healthcare staff vs linked national registers using routinely collected data) and one missing predictor. Differences in data collection between the development dataset (linked national registers) and the validation dataset (clinician-recorded assessments) did not materially affect discrimination, which remained good. This suggests that the model captures stable relative risk relationships that generalise across settings with different data capture processes, supporting its transportability across healthcare systems and modes of data collection.

The good performance in external validation is in keeping with the OxSATS development: a simple, transparent model was preferred, and predictors were defined in ways expected to generalise to different national settings. Other tools developed using this overall approach have shown similarly strong discrimination performance in external validation. For example, the Oxford Mental Illness and Suicide (OxMIS) tool, developed with Swedish data to model suicide risk in individuals with severe mental health problems,^12^ had an AUC of 0.70 (95% CI 0.69 to 0.71) in an external validation using Finnish data^13^ as compared with 0.71 (95% CI 0.66 to 0.75) in Swedish data. A recent review found that suicide risk prediction models show variable reduction in predictive performance when moving from internal to external validation, ranging from approximately 1% to 25% loss depending on the model used (derived from Seyedsalehi *et al*^14^).

Despite these differences, OxSATS remains distinctive in the UK context as a model specifically developed and externally validated for suicide outcomes following self-harm, rather than for composite outcomes. In contrast, most existing tools have focused on combined outcomes of self-harm and suicide and have not reported calibration.^14^

In general, there are few studies that directly compare structured risk assessment tools with clinical judgement for the prediction of suicide and self-harm outcomes.^15^ In the largest study to date comparing clinician judgement with structured assessments, clinician prediction of suicide death was not demonstrably above chance, with an AUC of 0.57 (95% CI 0.36 to 0.73).^16^ Risk models tools could therefore augment clinical judgement, supporting transparency and reproducibility in clinical assessment and raise the ceiling of expertise by anchoring assessment in empirically derived models based on combining multiple risk markers. This is particularly relevant for services where access to trained mental health specialists is limited. In such settings, structured risk tools may be particularly useful by providing a consistent framework to support risk assessment alongside routine clinical care.

Examination of hybrid decision-making frameworks that formally combine probabilistic prediction with clinical judgement should be a priority for future research. One next step for research is to evaluate clinician accuracy both in isolation and when using statistical risk estimates.^15^ Such designs would allow separation of baseline clinical judgement, the incremental value added by risk models and potential interaction effects, such as whether structured risk estimates improve calibration, consistency or discrimination in clinicians’ assessments. This approach moves beyond simplistic dichotomies between ‘tools versus clinicians’ and treats prediction as a sequential and collaborative process that more closely reflects real-world clinical workflows.

Good discrimination despite differences in predictor definitions and prevalence likely also reflects the stability of relative risk ordering across settings. Key predictors such as age, sex, and prior self-harm are consistently associated with suicide risk, allowing the model to rank individuals accurately even when measurements differ. This highlights the distinction between discrimination, which depends on relative risk, and calibration, which is sensitive to absolute event rates and predictor distributions.

Using risk tools may also allow for more efficient resource allocation by making more explicit how various factors contribute to risk. There is an important difference, however, between the performance of a risk model, which is concerned with accuracy, and the decision about resource allocation, which will need to consider a range of individual and wider ethical and service-related factors. A health economic analysis has found that the OxMIS tool was cost-saving when integrated into clinician assessment of suicide risk in an English setting^17^—similar assessments of the OxSATS model are warranted. In the US, studies have found that positive predictive values lower than 1% for self-harm and lower than 0.1% for suicide attempts were associated with cost-effectiveness.^18^ To our knowledge, the accuracy of unaided clinician prediction of suicide following self-harm has not been investigated in the UK, representing an important gap in the evidence base.

### Challenges of external validation

Our study demonstrates several challenges in externally validating a risk model in a new country. A first challenge is that some predictor definitions differed. In the current investigation, mental health variables were defined as problems that clinicians judged as directly related to the presenting self-harm, while in the OxSATS development sample they were defined as diagnosed psychiatric disorders at any time prior to the index self-harm. This led to a lower prevalence of these variables in the Oxford Monitoring System for Self-harm data, and their effect was less strong but in a similar direction in the final multivariable model. Despite this, discriminative performance was good, and reliance on proxy predictors means that the reported performance is likely to present a conservative estimate of how well OxSATS performs.^19^ The cohort was derived from a single hospital, which may limit generalisability within England, as local service provision, case-mix and referral pathways may differ from other regions.

Another challenge was that one predictor was missing in the English data—current psychotropic medication use—which was an independent predictor in the original OxSATS model. Sensitivity analyses showed that assuming different prevalences for this predictor affected calibration performance. We assessed this by systematically varying the assumed prevalence and estimating calibration using bootstrap resampling, which allowed uncertainty arising from both sampling variability and assumptions about the missing predictor to be reflected in the performance estimates.

In addition to the effect of this missing predictor, differences in the prevalence of other predictors between datasets, such as medication overdose and overnight admission, may also have contributed to between-cohort differences in predicted risk and model performance. Complete-case analysis and analysis of imputed data produced comparable results, suggesting that missing data were unlikely to have materially affected the findings. Nevertheless, as with all routinely collected clinical data, some characteristics were likely under-recorded.

We found that there was a lower outcome prevalence in the English data (0.6%) compared with the Swedish development sample (1.0%). This may reflect differences in case-mix, service provision, referral pathways, and outcome ascertainment, as the sample was derived from a single hospital and may not fully represent national patterns. For example, variation in thresholds for psychosocial assessment, local clinical practices and linkage to mortality data may influence observed event rates. These factors should be considered when interpreting calibration. This provides an explanation for why the OxSATS model initially overpredicted risk of suicide after self-harm. Since recalibration lowered the predicted probabilities, the original OxSATS binary decision cut-offs are no longer directly applicable and would require local re-evaluation by the clinician or service that apply the tool. In addition, inclusion of deaths of undetermined intent (ICD-10: Y10–Y34) in sensitivity analyses reflects a trade-off between sensitivity and specificity. While this approach reduces underascertainment of suicide, it may introduce misclassification by including some deaths not attributable to intentional self-harm (eg, accidental overdose).

## Conclusions

We have externally validated the OxSATS model in an English clinical setting, where it demonstrated good predictive performance, while also identifying a range of methodological challenges in validating risk models in new healthcare systems. These challenges include handling missing predictors and the need for model recalibration. Implementation strategies for prediction models could consider external validation with transparent reporting of discrimination and calibration.

## Supplementary material

10.1136/bmjment-2026-302568 online supplemental file 1

10.1136/bmjment-2026-302568 online supplemental file 2

## Data Availability

No data are available.
