# Supplementary material for: External validation, recalibration and updating of the OxSATS risk model for suicide after self-harm in England
Source: BMJ Ment Health. 2026 Sep 12;29(1):e302568. doi: 10.1136/bmjment-2026-302568 (PMC13583793; doi:10.1136/bmjment-2026-302568)
Supplement: Supplementary file 2 [file bmjment-29-1-s001.docx]

**SUPPLEMENT**

[Table S1. Missing data distribution	2](#_Toc229604451)

[Table S2. Demographic and clinical characteristics of the study cohort (by sex)	3](#_Toc229604452)

[Table S3. Distribution of outcomes with different definitions	4](#_Toc229604453)

[Table S4. Sensitivity analyses across outcome definitions, episode selection approaches, and follow-up periods on complete case data	5](#_Toc229604454)

[Table S5. Sensitivity analyses across outcome definitions, episode selection approaches, and follow-up periods on imputed data	6](#_Toc229604455)

[Table S6. Prevalence of OxSATS predictors in the Oxford Monitoring System for Self-harm dataset, overall and stratified by whether individuals commit suicide within 1 year	7](#_Toc229604456)

[Table S7. Mean calibration metrics when randomly assigning the missing predictor as 1 or 0 to individuals in the study population following a Bernoulli distribution with p=0.557, compared to the main analysis	8](#_Toc229604457)

[Table S8. OxSATS model parameters and formula for probability of committing suicide after self-harm presentation	9](#_Toc229604458)

[Figure S1. Calibration of the original OxSATS tool in the Oxford Monitoring System for Self-harm data	10](#_Toc229604459)

[Figure S2. Calibration of OxSATS model in the Oxford Monitoring data when receipt of any psychotropic medication is changing from 0 to 1.	11](#_Toc229604460)

## Table S1. Missing data distribution

The total dataset contained 31,649 total records, one per admission with self-harm. A given individual could have one or more admissions. The missing data is reported as number and proportion of admission records missing.

| Variable name | No. missing | % missing |
| --- | --- | --- |
| Age, years | 0 | 0.0 |
| Female | 24 | 0.1 |
| Problems* with alcohol | 11,190 | 35.4 |
| Problems* with other substances | 8,635 | 27.3 |
| Self-harm type   - Index self-harm: overdose with psychotropic medication - Index self-harm: hanging, strangulation, or suffocation | 336 | 1.1 |
| Lifetime history of self-harm prior to index | 5,668 | 17.9 |
| History of self-harm in last 12 months prior to index | 5,668 | 17.9 |
| Index self-harm: overnight admission | 5 | 0.0 |
| Problems* with mental health | 8,637 | 27.3 |

Note: * Problems = “the problem which may have precipitated the episode of SH or is associated with it in the clinician’s judgment.”

## Table S2. Demographic and clinical characteristics of the study cohort (by sex)

| **Characteristic** | **Men** | **Women** | **Total** |
| --- | --- | --- | --- |
| N (persons) | 6,493 | 9,625 | 16,130 |
| Age, years (mean ± SD) | 33.9 (±15.1) | 30.0 (±15.1) | 31.6 (±15.2) |
| Age, years (median, IQR) | 30.0 (22.0-42.0) | 25.0 (19.0-39.0) | 27.0 (20.0-40.0) |
| Problems* with alcohol (AUD) | 1,317 (20.3%) | 1,304 (13.5%) | 2,622 (16.3%) |
| Problems* with other substances (SUD) | 660 (10.2%) | 459 (4.8%) | 1,119 (6.9%) |
| Index SH: overdose with psychotropic medication | 1,826 (28.1%) | 2,913 (30.3%) | 4,740 (29.4%) |
| Index SH: hanging/strangulation/suffocation | 180 (2.8%) | 74 (0.8%) | 254 (1.6%) |
| Lifetime history of self-harm | 2,473 (38.1%) | 4,550 (47.3%) | 7,030 (43.6%) |
| History of self-harm in last 12 months | 1,465 (22.6%) | 2,817 (29.3%) | 4,288 (26.6%) |
| Index SH: overnight admission | 4,965 (76.5%) | 7,684 (79.8%) | 12,658 (78.5%) |
| Problems* with mental health | 941 (14.5%) | 1,404 (14.6%) | 2,350 (14.6%) |
| Suicide within 1 year (primary, incl. Y10-Y34) | 73 (1.1%) | 33 (0.3%) | 106 (0.7%) |
| Suicide within 1 year (alternative, excl. Y10-Y34) | 55 (0.8%) | 22 (0.2%) | 77 (0.5%) |
| Suicide within 3 years (primary, incl. Y10-Y34) | 106 (1.6%) | 58 (0.6%) | 164 (1.0%) |
| Suicide within 3 years (alternative, excl. Y10-Y34) | 81 (1.2%) | 39 (0.4%) | 120 (0.7%) |
| Follow-up months (mean ± SD) | 11.8 (±1.3) | 11.9 (±0.9) | 11.9 (±1.1) |

Note: The columns do not sum up due to missing sex variable for some patients. * Problems = “the problem which may have precipitated the episode of SH or is associated with it in the clinician’s judgment.

## Table S3. Distribution of outcomes with different definitions

The primary outcome definition included deaths coded as intentional self-harm or undetermined intent (ICD-10 X60–X84, Y10–Y34), while the alternative definition included intentional self-harm deaths only (ICD-10 X60–X84). Inclusion of deaths of undetermined intent increased the number of suicide outcomes by 27% at both 1-year and 3-year follow-up.

|  | Description | No. | Proportion of outcomes |
| --- | --- | --- | --- |
|  | Total eligible episodes | 16130 | — |
|  |  |  |  |
| 1 year | Suicides (primary, incl. Y10-Y34) | 106 | 0.7% |
|  | Suicides (alternative, excl. Y10-Y34) | 77 | 0.5% |
|  | Records with any Y10-Y34 code | 79 | 0.5% |
|  | Additional suicides from Y10-Y34 (primary vs alternative) | 29 | 27% of primary outcome |
|  |  |  |  |
| 3 year | Suicides (primary, incl. Y10-Y34) | 164 | 1.0% |
|  | Suicides (alternative, excl. Y10-Y34) | 120 | 0.7% |
|  | Records with any Y10-Y34 code | 79 | 0.5% |
|  | Additional suicides from Y10-Y34 (primary vs alternative) | 44 | 27% of primary outcome |
|  |  |  |  |

## Table S4. Sensitivity analyses across outcome definitions, episode selection approaches, and follow-up periods on complete-case data

Model discrimination remained broadly consistent across analyses, with c-index values ranging from 0.71 to 0.76. Calibration analyses showed persistent overprediction across all scenarios, with O/E ratios below 1. Results were similar when using random versus first self-harm episode selection approaches. Restricting outcomes to deaths of undetermined intent (Y10–Y34) was associated with slightly higher discrimination but lower O/E ratios compared with analyses including both intentional self-harm and undetermined intent deaths (X60–X84, Y10–Y34).

| Follow-up period | ICD-10 codes for outcome | Episode selection | N events | C-index (95% CI) | O/E (95% CI) |
| --- | --- | --- | --- | --- | --- |
|  |  |  |  |  |  |
| 1 year | X60–X84, Y10–Y34 | Random self-harm episode | 68 | 0.73 (0.68, 0.79) | 0.56 (0.43, 0.70) |
|  | X60–X84, Y10–Y34 | First self-harm episode | 61 | 0.74 (0.68, 0.79) | 0.52 (0.39, 0.65) |
|  | Y10–Y34 | Random self-harm episode | 53 | 0.76 (0.70, 0.81) | 0.43 (0.32, 0.56) |
|  | Y10–Y34 | First self-harm episode | 48 | 0.75 (0.70, 0.81) | 0.41 (0.30, 0.53) |
|  |  |  |  |  |  |
| 3 years | X60–X84, Y10–Y34 | Random self-harm episode | 108 | 0.71 (0.66, 0.75) | 0.46 (0.38, 0.55) |
|  | X60–X84, Y10–Y34 | First self-harm episode | 94 | 0.71 (0.66, 0.76) | 0.42 (0.34, 0.50) |
|  | Y10–Y34 | Random self-harm episode | 82 | 0.75 (0.70, 0.79) | 0.35 (0.28, 0.43) |
|  | Y10–Y34 | First self-harm episode | 72 | 0.74 (0.69, 0.79) | 0.32 (0.25, 0.40) |
|  |  |  |  |  |  |

## Table S5. Sensitivity analyses across outcome definitions, episode selection approaches, and follow-up periods on imputed data

To assess the potential effect of missing data, we conducted sensitivity analyses using multiple imputation by chained equations. Missing data were imputed and the same modelling procedure was repeated across several outcome definitions, including suicide deaths only (X60–X84) and suicide plus deaths of undetermined intent (X60–X84, Y10–Y34), using both random and first self-harm episode selection approaches and 1-year and 3-year follow-up periods.

| Follow-up period | ICD-10 codes included in outcome definition | Episode selection | N events | C-index (95% CI) | O/E (95% CI) |
| --- | --- | --- | --- | --- | --- |
|  |  |  |  |  |  |
| 1 year | X60–X84, Y10–Y34 | Random self-harm episode | 106 | 0.70 (0.66, 0.75) | 0.57 (0.47, 0.69) |
|  | X60–X84, Y10–Y34 | First self-harm episode | 92 | 0.71 (0.66, 0.75) | 0.51 (0.41, 0.62) |
|  | Y10–Y34 | Random self-harm episode | 77 | 0.73 (0.68, 0.78) | 0.42 (0.32, 0.51) |
|  | Y10–Y34 | First self-harm episode | 68 | 0.72 (0.67, 0.77) | 0.38 (0.29, 0.47) |
| 3 years | X60–X84, Y10–Y34 | Random self-harm episode | 164 | 0.70 (0.66, 0.73) | 0.47 (0.40, 0.54) |
|  | X60–X84, Y10–Y34 | First self-harm episode | 146 | 0.69 (0.65, 0.73) | 0.43 (0.36, 0.50) |
|  | Y10–Y34 | Random self-harm episode | 120 | 0.72 (0.68, 0.76) | 0.34 (0.28, 0.40) |
|  | Y10–Y34 | First self-harm episode | 106 | 0.71 (0.66, 0.75) | 0.31 (0.25, 0.37) |
|  |  |  |  |  |  |

## Table S6. Prevalence of OxSATS predictors in the Oxford Monitoring System for Self-harm dataset, overall and stratified by whether individuals commit suicide within 1 year

|  | **Participants without suicide within 1 year (N=16024)** | **Participants with suicide within 1 year (N=106)** | **All participants (N=16130)** |
| --- | --- | --- | --- |
| **Age, years: Mean (SD)** | 31.5 (±15.2) | 41.6 (±17.1) | 31.6 (±15.2) |
| **Female** | 9,592 (59.9%) | 33 (31.1%) | 9,625 (59.7%) |
| **Problems* with alcohol** | 2,602 (16.2%) | 20 (18.9%) | 2,622 (16.3%) |
| **Problems* with other substances** | 1,116 (7.0%) | 3 (2.8%) | 1,119 (6.9%) |
| **Index self-harm: overdose with psychotropic medication** | 4,700 (29.3%) | 40 (37.7%) | 4,740 (29.4%) |
| **Index self-harm: hanging, strangulation, or suffocation** | 251 (1.6%) | 3 (2.8%) | 254 (1.6%) |
| **Lifetime history of self-harm prior to index** | 6,981 (43.6%) | 49 (46.2%) | 7,030 (43.6%) |
| **History of self-harm in last 12 months prior to index** | 4,253 (26.5%) | 35 (33.0%) | 4,288 (26.6%) |
| **Index self-harm: overnight admission** | 12,572 (78.5%) | 86 (81.1%) | 12,658 (78.5%) |
| **Problems* with mental health** | 2,326 (14.5%) | 24 (22.6%) | 2350 (14.6%) |

Note: * Problems = “the problem which may have precipitated the episode of SH or is associated with it in the clinician’s judgment.”

## Table S7. Mean calibration metrics when randomly assigning the missing predictor as 1 or 0 to individuals in the study population following a Bernoulli distribution with p=0.557, compared to the main analysis

|  | **Main analysis** | **Mean of 1000 simulations** |
| --- | --- | --- |
| **O:E (95% CI)** | 0.56 (0.43, 0.70) | 0.52 (0.52, 0.53) |
| **Slope** | 1.11 (0.80, 1.44) | 0.91 (0.79, 1.03) |

## Table S8. OxSATS model parameters and formula for probability of committing suicide after self-harm presentation

|  |  | **Parameter (β)** | **95% confidence interval** | |
| --- | --- | --- | --- | --- |
|  | **General demographics** | | | |
|  | (Age at index/10)^-2^ | -4.20 | -5.32 | -3.08 |
|  | Sex, female | -0.70 | -0.87 | -0.53 |
|  | **Substance misuse** | | | |
|  | Current or lifetime alcohol use disorder (excluding alcohol intoxication) | -0.03 | -0.22 | 0.17 |
|  | Current or lifetime drug use disorder (including drug intoxication) | 0.31 | 0.12 | 0.50 |
|  | **Treatment in the past three months** | | | |
|  | Any psychotropic medication | 0.76 | 0.52 | 1.01 |
|  | **History of self-harm** | | | |
|  | Any psychotropic medication overdose | 0.42 | 0.02 | 0.82 |
|  | Hanging, strangulation, or suffocation | 0.97 | 0.35 | 1.59 |
|  | History of self-harm in the last 12 months prior to index, 1+ vs none | 0.31 | 0.07 | 0.55 |
|  | Overnight admission | 0.57 | 0.38 | 0.75 |
|  | **Mental health in the past 12 months** | | | |
|  | Any psychiatric disorder except substance use disorders | 0.51 | 0.31 | 0.70 |
|  | **Other parameters in the model – original model** | | | |
|  | Intercept | -6.49 | -6.79 | -6.20 |
|  | Shape parameter | 0.60 | 0.55 | 0.65 |
| **Recalibrated model** | **Other parameters in the model – recalibrated model** | | | |
|  | Intercept | 3.58 | 0.67 | 6.48 |
|  | Multiplier for linear predictor (original intercept + sum of risk factors multiplied by their parameters) | -1.70 | -2.35 | -1.05 |
|  | Shape parameter | 0.65 | 0.52 | 0.83 |

Binary risk factors are coded as 1 if present and 0 if absent

In the original OxSATS tool, the risk for death by suicide within *t* months of presentation to specialist service with self-harm is calculated by the following formula:

$$\Pr\left( suicide within t months \right)=1-exp(-\exp\left( linpred \right)t^{0.60})$$

Where $linpred= -6.49+ \sum\beta*Risk Factor$ and *t* = 6 or 12

In the recalibrated model, the predicted probability of death by suicide within t **days** following self-harm is given by:

$$\Pr\left( suicide within t days \right)=1-{\exp\left( -\left[ \frac{t}{\exp\left( 3.58 - 1.70 \times linpred \right)} \right] \right)}^{0.65}$$

Where $linpred= -6.49+ \sum\beta*Risk Factor$ and *t* = 182 or 365

For a simplified reference profile with *linpred* = −6.49, the survivor function is:

$$S_{\mathrm{ref}}={\exp\left( -\left[ \frac{t}{\exp\left( 3.58-1.70 \times(-6.49) \right)} \right] \right)}^{0.65}$$

At one year this is $S_{ref}\left( 365 \right)\approx0.997$

## Figure S1. Calibration of the original OxSATS tool in the Oxford Monitoring System for Self-harm data

## Figure S2. Calibration of OxSATS model in the Oxford Monitoring data when receipt of any psychotropic medication is changing from 0 to 1.
